# Phase-diagram-guided method for growth of a large crystal of glycoside hydrolase family 45 inverting cellulase suitable for neutron structural analysis

**DOI:** 10.1107/S0909049513020943

**Published:** 2013-09-25

**Authors:** Akihiko Nakamura, Takuya Ishida, Shinya Fushinobu, Katsuhiro Kusaka, Ichiro Tanaka, Koji Inaka, Yoshiki Higuchi, Mika Masaki, Kazunori Ohta, Satoshi Kaneko, Nobuo Niimura, Kiyohiko Igarashi, Masahiro Samajima

**Affiliations:** a Graduate School of Agricultural and Life Sciences, The University of Tokyo, 1-1-1 Yayoi, Bunkyo-ku, Tokyo 113-8657, Japan; b Frontier Research Center for Applied Atomic Sciences, Ibaraki University, 164-1 Shirakita, Tokai-mura, Naka-gun, Ibaraki 319-1106, Japan; c Maruwa Foods and Biosciences, 170-1 Tsutsui, Yamatokouriyama, Nara 639-1123, Japan; d Department of Life Science, Graduate School of Life Science, University of Hyogo and Himeji Institute of Technology, 3-2-1 Koto, Kamigori-cho, Ako-gun, Hyogo 678-1297, Japan; e Japan Aerospace Exploration Agency, Tsukuba Space Center, 2-1-1 Sengen, Tsukuba, Ibaraki 305-8505, Japan; f National Food Research Institute, 1-2-12 Kannondai, Tsukuba, Ibaraki 304-8642, Japan

**Keywords:** cellulase, neutron protein crystallography, crystallization phase diagram

## Abstract

Neutron protein crystallography (NPC) is a powerful tool for determining the hydrogen position and water orientation in proteins, but a much larger protein crystal is needed for NPC than for X-ray crystallography, and thus crystal preparation is a bottleneck. To obtain large protein crystals, it is necessary to know the properties of the target protein in the crystallization solution. Here, a crystal preparation method of fungal cellulase *Pc*Cel45A is reported, guided by the phase diagram. Nucleation and precipitation conditions were determined by sitting-drop vapor diffusion. Saturation and unsaturation conditions were evaluated by monitoring crystal dissolution, and a crystallization phase diagram was obtained. To obtain a large crystal, crystallization solution was prepared on a sitting bridge (diameter = 5 mm). Initial crystallization conditions were 40 µl of crystallization solution (40 mg ml^−1^ protein with 30.5% 3-methyl-1,5-pentanediol in 50 m*M* tris-HCl pH 8.0) with a 1000 µl reservoir (61% 3-methyl-1,5,-pentanediol in 50 m*M* tris-HCl pH 8.0) at 293 K. After the first crystal appeared, the concentration of precipitant in the reservoir solution was reduced to 60% to prevent formation of further crystals. Finally, we obtained a crystal of 6 mm^3^ volume (3 mm × 2 mm × 1 mm), which was suitable for neutron diffraction.

## Abstract 2

The crystallization-phase-diagram-guided method is effective for growing large protein crystals for neutron protein crystallography.

## 1. Introduction

Three-dimensional structures of many proteins have been solved in order to understand the reaction mechanisms of enzymes and interactions of proteins. Generally, proteins interact with water molecules and ligands *via* hydrogen bonds, and in many cases, such as hydrolytic enzymes, a water molecule is one of the substrates. Therefore, experimental methods to locate hydrogen are important for a better understanding of protein functions. Neutron protein crystallography (NPC) is a powerful technique for determining the positions of hydrogen and the orientation of water molecules in the crystal structure of proteins (Niimura & Podjarny, 2011 ▶). Nevertheless, NPC has several drawbacks. For example, although only a few hours (or minutes) of data collection using small crystals are enough for X-ray protein crystallography, NPC requires at least a few weeks (up to several months) and a large crystal (>1 mm^3^) to obtain a complete set of data. While the performance of instruments, such as neutron beam generators and detectors, has been improving (Tanaka *et al.*, 2010 ▶; Blakeley *et al.*, 2010 ▶; Kovalevsky *et al.*, 2010 ▶), techniques for preparing large protein crystals are still insufficiently developed.

Endoglucanase (EC 3.2.1.4) belonging to glycoside hydrolase (GH) family 45 in the carbohydrate-active enzymes (CAZy) database (Cantarel *et al.*, 2009 ▶), formally known as family K cellulase, is an enzyme produced by various organisms. This family has been divided into three sub-families (A, B and C) from the phylogenetic analysis (Igarashi *et al.*, 2008 ▶), whereas the detailed character has been demonstrated only for the sub-family A cellulase from *Humicola insolens* (Cel45A). This enzyme has a pair of carboxylic residues located in the substrate-binding cleft, and it hydrolyzes cellulose *via* an inverting mechanism (Davies *et al.*, 1995 ▶), in which one of the carboxylic residues acts as a general base and the other as a general acid. The general acid residue proton­ates the glycosidic oxygen in the substrate, while the general base residue activates the nucleophilic water molecule, which attacks the anomeric carbon (Vuong & Wilson, 2010 ▶). Although, in many cases, enzymes belonging to the same family have similar folds and catalytic residues, we recently discovered that Cel45A from the basidiomycete *Phanerochaete chrysosporium* (*Pc*Cel45A, sub-family C) has only a glutamic acid residue at the appropriate position for the general acid and no residue that has been assigned as a general base. Nevertheless, *Pc*Cel45A has hydrolytic activity (Igarashi *et al.*, 2008 ▶). For several inverting GH family members, biochemical and structural analyses have not given unambiguous results regarding the general base residue or the reaction mechanism (Vuong & Wilson, 2010 ▶). Therefore, we considered that NPC analysis of *Pc*Cel45A would give new insights not only into the reaction mechanism of this enzyme but also into the diversity of inverting GH mechanisms. For this purpose, a key issue is the preparation of a sufficiently large protein crystal. In this paper, we describe a phase-diagram-guided method for growth of a large crystal of *Pc*Cel45A suitable for NPC.

## 2. Materials and methods

### 2.1. Enzyme production

The gene encoding *Pc*Cel45A was inserted into pPicZα-A vector (Invitrogen) between the XhoI and NotI sites. A methylotrophic yeast, *Pichia pastoris* strain KM71H (Invitrogen), was transformed with the vector, and transformants were selected as described earlier (Igarashi *et al.*, 2008 ▶). The recombinant enzyme was produced on a 5 l scale in a mini-jar fermenter (TSC-M5L; Takasugi Seisakusho). Seed solution prepared by incubation of the selected transformant at 303 K with 10 ml of YPD medium was inoculated in 2 l of modified basal salt medium (Cregg, 2010 ▶) adjusted to pH 5.0 with 10% ammonium solution at 300 K. The pH of the medium was fixed at 5.0 by addition of 10% ammonium solution during the incubation. After one and a half days of incubation following the inoculation of seed culture, feeding of 50% glycerol (180 g) was started, and protein production was induced by adding 100% methanol under regulation by a dissolved oxygen controller. After 50 h of induction, the culture medium was collected by centrifugation (30 min at 3000*g* and 30 min at 10000*g*). The cell-free culture medium was passed through a 100 kDa cut-off ultrafilter to remove polymer components, and stored at 253 K. The hydrolytic activity towards 0.1% phosphoric-acid-swollen cellulose was measured in terms of production of reducing sugar in 100 m*M* sodium acetate buffer pH 5.0 at 303 K. Protein concentration was measured with protein assay kits (Bio-rad) according to the manufacturer’s instructions.

### 2.2. Enzyme purification

Ammonium sulfate was added to 200 ml of culture medium to give a final concentration of 1 *M*. The solution was injected into a phenyl-Toyopearl 650S column (70 ml of carrier) equilibrated with 20 m*M* sodium acetate buffer containing 1 *M* ammonium sulfate (pH 5.0). The protein was eluted with a 280 ml reverse gradient to 20 m*M* sodium acetate buffer (pH 5.0). The fractions containing the recombinant protein were collected, equilibrated against 20 m*M* tris-HCl buffer (pH 8.0) and applied to a SuperQ-Toyopearl 650S column (100 ml of carrier) equilibrated with the same buffer. The protein was eluted from the column with a linear gradient from 0 to 0.08 *M* NaCl in 1000 ml. The protein solution was desalted and injected into a DEAE-Toyopearl 650S column (150 ml of carrier) equilibrated with 20 m*M* tris-HCl buffer (pH 8.0). The protein was eluted from the column with a linear gradient from 0 to 0.06 *M* NaCl in 900 ml. The protein solution was concentrated and equilibrated with 20 m*M* tris-HCl buffer (pH 8.0) and stored at 277 K. The purity was confirmed by SDS-PAGE and basic native-PAGE (Mini-protean tetra cell; Bio-rad), as well as HPLC (LC-2000 series; Jasco) on an anion-exchange column (TSKgel DEAE-5PW; Tosho). Size monodispersity was confirmed by dynamic light scattering (Dynapro DLS; Wyatt).

### 2.3. Determination of crystal phase diagram

Ammonium sulfate, lithium sulfate, ethanol, propanol, 2-methyl-2,4-pentanediol, 3-methyl-1,5-pentanediol, polyethylene glycol 3350 and 8000 was tested as precipitant. Used buffers were 50 m*M* of sodium citrate (pH 3.0), sodium acetate (pH 4.0 to 5.5), 2-morpholinoethanesulfonic acid (pH 6.0), 4-(2-hydroxyethyl)-1-piperazineethanesulfonic acid (pH 7.0), tris-HCl (pH 7.5 to 8.0), *N*-cyclohexyl-2-aminopropanesulfonic acid (pH 9.0) and *N*-cyclohexyl-3-aminopropanesulfonic acid (pH 10.0). To make a crystal phase diagram, combinations of 3-methyl-1,5-pentanediol (0 to 100% in 10% steps) and *Pc*Cel45A (0 to 80 mg ml^−1^ in 10 mg ml^−1^ steps) in 50 m*M* tris-HCl (pH 8.0) were tested. Aliquots of 100 µl of each reservoir solution were prepared in wells of 96-well plates for sitting-drop vapor diffusion (Greiner). Aliquots of 1 µl of each concentration of protein solution were mixed with an equal amount of reservoir solutions in sample wells. Three drops were prepared for each condition, and plates were incubated at 293 K. The condition of wells was checked appropriately, and crystal formation was finally checked after a month. Conditions that yielded crystals in the wells were considered to be nucleation conditions, while conditions that yielded amorphous precipitates were considered to be precipitation conditions. Saturation or unsaturation was discriminated by observing the dissolution of an added small crystal. To refine the nucleation phase, additional points near the border between the nucleation phase and saturation phase were checked by the same method. The saturation-limit concentration of protein in 60% 3-methyl-1,5-pentanediol was evaluated by measurement of the supernatant in the well after growth of a large crystal.

### 2.4. Growth of a large crystal

Initially, 1000 µl of reservoir solution consisting of 63% 3-methyl-1,5-pentanediol containing 50 m*M* tris-HCl pH 8.0 was prepared in a 24-well plate (Greiner). Sitting bridges (20 µl volume) were placed in the wells, and 20 µl of reservoir solution and the same amount of 40 mg ml^−1^ *Pc*Cel45A were mixed on the bridges. The plate was incubated at 293 K and crystal generation was observed. When the first crystal appeared, the concentration of precipitant was diluted to 60% to prevent further nucleation, and incubation was continued for a month.

Next, the effects by increasing the protein concentration and scaling up the crystallization volume were examined separately. For the former, the same crystallization procedure as in the first trial was used, except that the protein concentration was increased to 80 mg ml^−1^ and the precipitant concentration was decreased to 61%. To scale up the crystallization volume, 5 ml of the same reservoir solution as in the first trial was prepared in a plastic case (diameter = 42 mm), in which a sample cup (diameter = 10 mm) was placed for drop crystallization. Then 200 µl aliquots of 40 mg ml^−1^ *Pc*Cel45A and reservoir solution were mixed in the cup, and incubation was carried out at 293 K. In both cases, other procedures (incubation, observation and dilution of precipitant) were the same as in the first trial.

Crystals were also prepared in D_2_O using the conditions with increasing protein concentration.

### 2.5. Crystal quality evaluation

The quality of a large crystal prepared as described above was evaluated by X-ray diffraction. The crystal prepared in D_2_O was packed in a quartz glass capillary (diameter = 3.5 mm, wall thickness = 0.01 mm) with 60% 3-methyl-1,5-pentanediol solution containing 2 mg ml^−1^ *Pc*Cel45A. X-ray diffraction measurements were recorded on an R-axis IV^++^ (Rigaku) at 293 K, and diffraction images were processed using *XDS* (Kabsch, 2010 ▶).

## 3. Results and discussion

### 3.1. Enzyme production

In order to obtain large protein crystals, large-scale production of protein is essential. Therefore, selection of a suitable host and production method is important. Because the present enzyme was originally from a basidiomycete and has many disulfide bridges in the structure, the yeast *Pichia pastoris* was chosen as a host for *Pc*Cel45A protein production (Cereghino & Cregg, 2000 ▶). A jar-fermenter was employed, as it is easy to maintain the desired pH of the culture, which is favorable for *P. pastoris* growth and enzyme stability.

**Figure 1:**
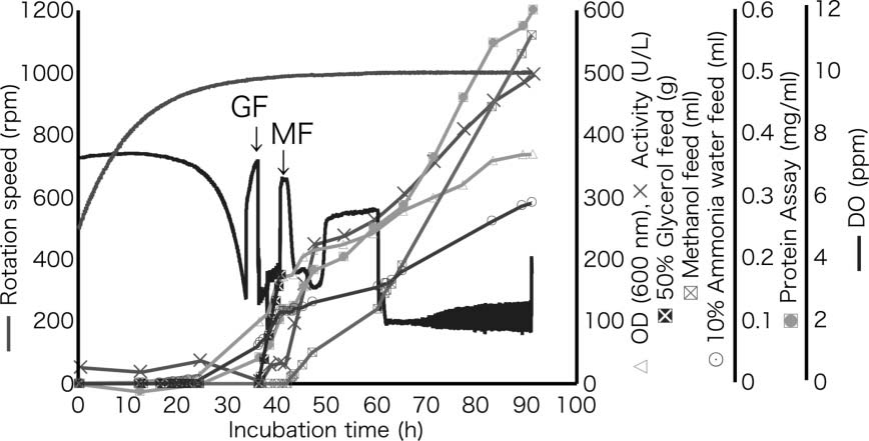
Production of recombinant *Pc*Cel45A by the methylotrophic yeast *P. pastoris* in jar-fermenter culture (GF: glycerol feed; MF: methanol feed).

The results of incubation are summarized in Fig. 1 ▶. The cell density of *P. pastoris* [optical depth (OD) 600 nm] reached 171 during glycerol feeding and further increased during methanol feeding (final OD 600 nm was 369). The pH and temperature of the culture were kept at 5.0 and 300 K, respectively. The pH was raised by addition of 10% ammonia water as necessary, and the total fed volume was 290 ml. Because the amount of ammonia water added was correlated with the increment of cell density, ammonium was presumably consumed by *P. pastoris* as a nitrogen source. The final methanol feed amount was 560 ml and the added amount was related to the protein concentration. The increases of activity and protein concentration were similar until 71 h, but towards the end of the incubation the increase of activity was smaller than that of protein concentration. This phenomenon indicates that inactive protein(s) was secreted in the culture. Hence, the incubation was stopped at 91 h. Although the protein concentration finally reached 0.6 mg ml^−1^, the *Pc*Cel45A concentration calculated from the activity (496 U/L at 91 h incubation) was 1.66 mg ml^−1^. This difference is due at least in part to resistance of *Pc*Cel45A to dying by the pigment in the protein assay kit. Finally, 2200 ml of culture medium, which was estimated to contain 3.6 g of *Pc*Cel45A, was collected.

### 3.2. Enzyme purification

High purity is very important for crystallization, especially for obtaining large crystals, because impurities in the crystallization solution slow crystal growth and also cause cracking of the crystals during growth due to the interactions between impurities and the crystal surface (Caylor *et al.*, 1999 ▶). Because the relative ratio of impurities in the crystallization solution is increased during the crystal growth, even a small amount of impurities can prevent the successful formation of large crystals.

**Figure 2:**
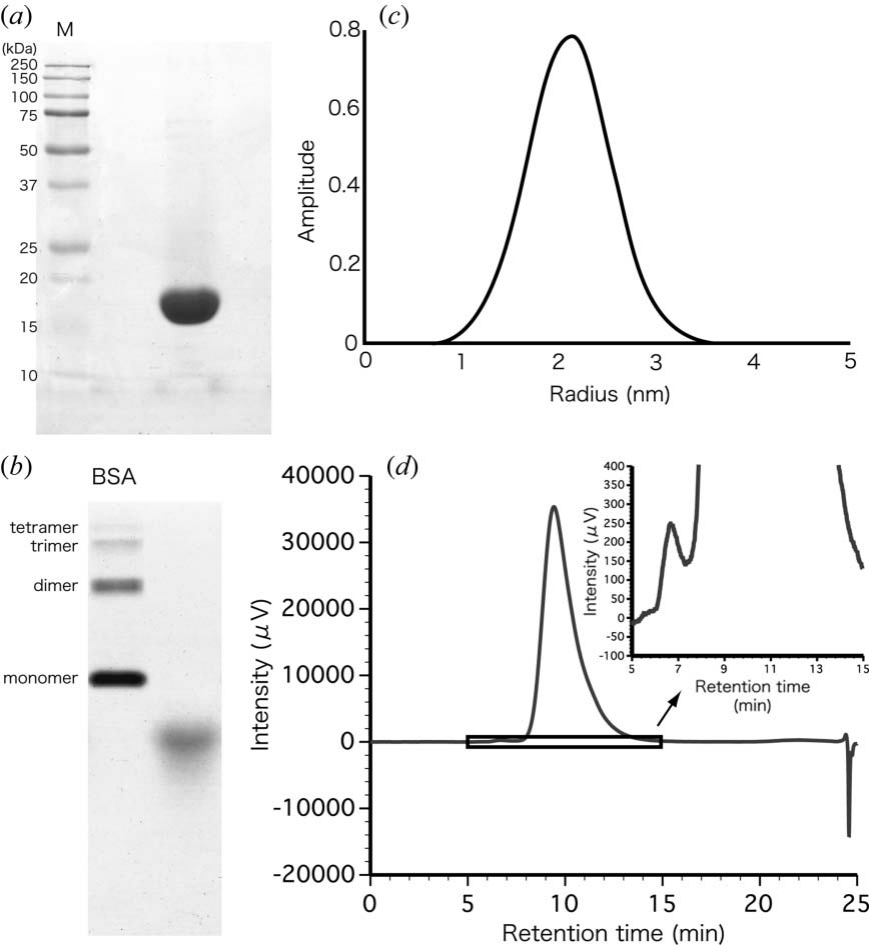
(*a*) SDS-PAGE of 40 µg of *Pc*Cel45A. (*b*) Native-PAGE of 80 µg of *Pc*Cel45A. (*c*) Result of dynamic light scattering of 40 mg ml^−1^ *Pc*Cel45A in 20 m*M* tris-HCl pH 8.0 at 293 K. (*d*) HPLC chromatogram of 80 µg of PcCel45A on an anion-exchange column.

After three steps of chromatography, we obtained 158 mg of enzyme from 200 ml of culture medium. SDS-PAGE of 40 µg of purified *Pc*Cel45A showed a single band (Fig. 2*a* ▶), and basic native PAGE of 80 µg also showed a single band (Fig. 2*b* ▶). These results indicate that contaminating proteins of similar size and isoelectric point to *Pc*Cel45A amounted to less than 0.25% (less than 0.1 µg contaminant per 40 µg of *Pc*Cel45A). The intensity distribution of dynamic light scattering is shown in Fig. 2(*c*) ▶. Purified *Pc*Cel45A showed a mono-modal size distribution in the protein solution with a polydispersity index of 0.04. The estimated radius and molecular mass were 2.1 ± 0.4 nm and 17.5 kDa, respectively. This result is consistent with the molecular mass calculated for the gene-encoded protein (18 kDa). To further confirm the purity, 80 µg of *Pc*Cel45A was subjected to HPLC with various salt gradients, and a single small peak of impurity was detected. The purity of the enzyme calculated from the ratio of peak areas was 99.9%. Therefore, the purified *Pc*Cel45A was considered suitable for crystallization (Bergfors, 1999 ▶).

### 3.3. Crystal phase diagram

**Figure 3:**
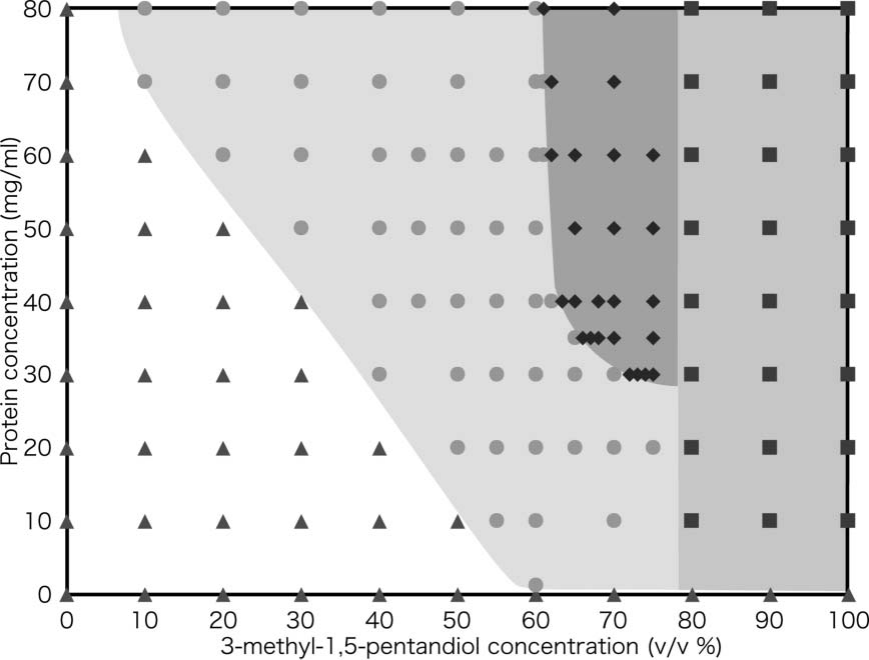
Crystallization phase diagram of *Pc*Cel45A in 50 m*M* tris-HCl buffer pH 8.0 at 293 K. Triangles: unsaturation; circles: saturation; diamonds: nucleation; squares: precipitation.

The crystal phase diagram (CPD), which consists of nucleation, precipitation (or isolation), saturation and un­saturation zones, was constructed as described above, and allowed us to identify suitable conditions of protein and precipitant concentrations for crystal growth and nucleation (Fig. 3 ▶). The nucleation zone lay in the range between 61% and 80% 3-methyl-1,5-pentanediol. The lower limit of protein concentration for nucleation was 30 mg ml^−1^ at 75% precipitant, and the enzyme precipitated at 80% or more of the precipitant. The saturation zone was large, and the lower limit of protein concentration at 60% precipitant was 1.2 mg ml^−1^. To obtain sufficient data for construction of the CPD, about 15 mg of *Pc*Cel45A was needed.

### 3.4. Growth of a large crystal

Because the amount of protein in a well is limited, a key factor in making a large crystal is control of nucleation. In other words, it is important to prevent multiple nucleation. We considered that this might be done by decreasing the precipitant concentration in the crystallization solution after formation of the first crystal. Since the CPD allows us to identify the lower limit of precipitant concentration for nucleation, it can be used to develop a strategy for managing the crystallization conditions.

**Figure 4:**
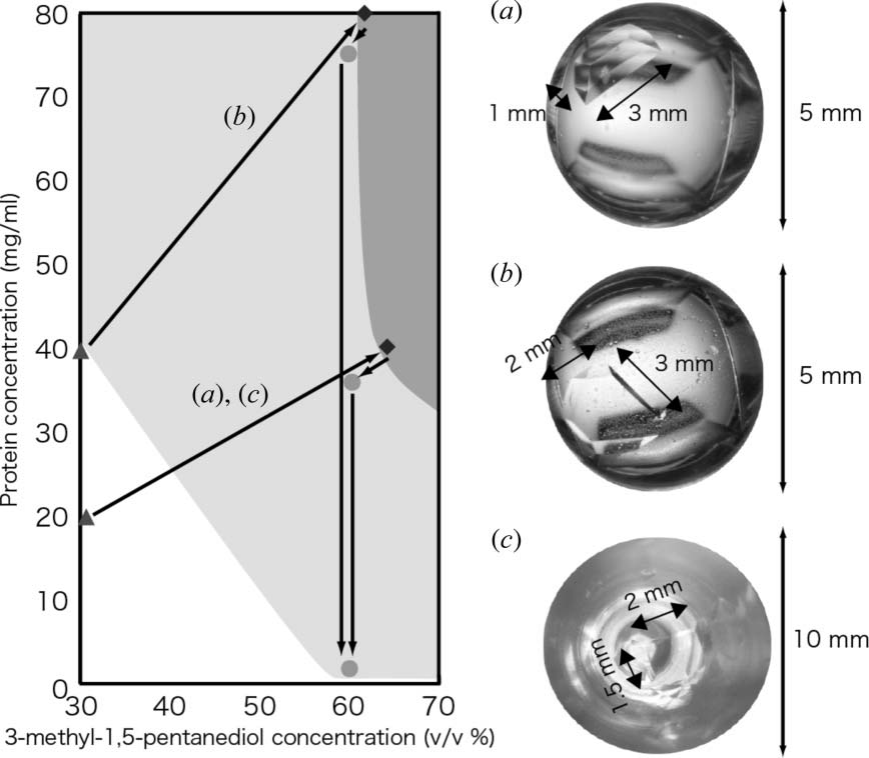
Crystallization strategy and crystal growth. (*a*) 0.8 mg protein/20 µl well. (*b*) 1.6 mg protein/20 µl well. (*c*) 8.0 mg protein/200 µl well.

Based on the above idea, we first examined direct reduction of the precipitant concentration in the crystallization solution by dilution after a crystal had formed. In this case, a crystal of about 3 mm^3^ volume was obtained from 0.8 mg of *Pc*Cel45A (20 µl of 40 mg ml^−1^ *Pc*Cel45A), and the protein concentration in the crystallization solution was decreased to 1.2 mg ml^−1^ (*i.e.* only 24 µg of enzyme remained in the liquid) (Fig. 4*a* ▶). This result indicated that the amount of protein in the well was not enough to form a larger crystal.

Next, we examined two different strategies, *i.e.* increasing the protein concentration and increasing the volume of the crystallization solution. The former method provided a crystal of about 6 mm^3^ volume (Fig. 4*b* ▶), but the latter resulted in formation of many small crystals (Fig. 4*c* ▶). One reason for the difference may be that the probability of nucleation per volume is constant, so the total number of nucleations will be greater in a large volume of crystallization solution than in a small volume. Thus, in the present case, control of the protein concentration was the key to success.

### 3.5. Crystal quality evaluation

Not only the size but also the quality of the crystal is an important factor for single-crystal structure analysis. For evaluation of the orientation of water molecules by NPC, a higher resolution than 2 Å is needed. Therefore, the quality of the obtained crystal was evaluated by X-ray diffraction analysis.

The results of X-ray diffraction analysis are summarized in Table 1 ▶. Although the highest-resolution limit was 1.63 Å because of the instrumental limitations of in-house X-ray instruments, *I*/σ of the highest shell was 21.75. In addition, the averaged mosaicity of the high-range data set was 0.058° estimated by *XDS*, and the Wilson *B*-factor of the merged data set was 10.6 Å^2^. Considering that the mosaicity estimated by *XDS* is typically low compared with that estimated by *Mosflm* or *HKL2000* (Mueller *et al.*, 2012 ▶; Remmerie *et al.*, 2008 ▶), these data still indicate that our CPD-guided crystallization method was effective in preparing a large crystal suitable for NPC.

## 4. Conclusion

*Pc*Cel45A was produced on a large scale by growing *P. pastoris* transfected with a vector encoding *Pc*Cel45A in a fermenter, and highly purified by means of three steps of chromatography. The crystal phase diagram of the enzyme was evaluated and the border between nucleation conditions and saturation conditions was clearly defined. This information was utilized to select a suitable crystallization strategy, and a large *Pc*Cel45A crystal with volume of 6 mm^3^ was grown. X-ray diffraction analysis confirmed that the quality of this crystal was sufficient for NPC. Thus, the CPD-guided crystallization method reported in this paper is effective for growing large protein crystals without the need for special equipment.

## Figures and Tables

**Table 1:** X-ray data collection statistics Numbers in parentheses are for the highest-resolution shell.

	Data set		
	Merged	High	Low
Space group	*P*2_1_2_1_2_1_		
Unit-cell parameters			
*a* (Å)	46.2		
*b* (Å)	59.1		
*c* (Å)	64.4		
X-ray source	Cu anode (50 kV, 100 mA)		
Wavelength (Å)	1.54		
Resolution (Å)	46.1–1.63 (1.67–1.63)	46.1–1.63 (1.73–1.63)	46.1–3.22 (3.41–3.22)
Total reflections	174337 (10841)	153723 (23370)	20236 (2582)
Unique reflections	23660 (1617)	22567 (3546)	3073 (459)
Completeness (%)	99.9 (99.7)	99.8 (98.7)	99.1 (94.8)
Multiplicity	7.4 (6.7)	6.8 (6.6)	6.6 (5.6)
〈*I*/*σ*(*I*)〉	34.94 (21.75)	33.65 (25.28)	34.66 (30.28)
*R*_sym_ (%)	–	4.7 (6.2)	4.6 (5.1)
*R*_merge_ (%)	4.1 (5.0)	–	–
Averaged mosaicity (°)	–	0.058	0.019
Wilson *B*-factor (Å^2^)	10.6	10.5	14.7
